# Beyond access: how heterogeneity of internet use differentially shapes cognitive function among Chinese older adults

**DOI:** 10.3389/fpubh.2026.1851159

**Published:** 2026-07-20

**Authors:** Rongrong Xiao, Hang Liang, Yifeng Liu, Zhihui Wei

**Affiliations:** 1 School of Social Development, Tianjin University of Technology, Tianjin, China; 2 School of Economics and Management, Hubei Innovation Research Center of Rural Social Management, Hubei University of Technology, Wuhan, China; 3 School of Humanities and Social Sciences, Nanjing Forestry University, Nanjing, China

**Keywords:** cognitive function, internet use, living arrangement, multidimensional internet use, older adults

## Abstract

**Background:**

The intersection of digitalization and population aging has positioned internet use and cognitive function among older adults as a salient public health concern. However, existing research has predominantly treated internet use as a binary variable, failing to adequately uncover the heterogeneous effects of usage behaviors. Moreover, the moderating role of living arrangement has received insufficient attention.

**Methods:**

This study analyzed two-wave panel data from the China Longitudinal Aging Social Survey (CLASS) in 2018 and 2020, comprising 6,800 older adults aged 65 and above, yielding 13,600 person-wave observations. Cognitive function was assessed using the Mini-Mental State Examination (MMSE), encompassing orientation, memory, and calculation. Internet use was operationalized as a four-dimensional construct, including internet adoption, use intensity, device operation proficiency, and function types. Two-way fixed effects models were employed to estimate the associations between multidimensional internet use and cognitive function, and to test the moderating role of living arrangement.

**Results:**

Internet adoption exerted a protective effect on overall cognitive function. Among continuous users, use intensity and instrumental us promoted cognitive improvement, whereas device operation proficiency was negatively associated with cognitive function. These effects exhibited significant heterogeneity across cognitive subdomains, including orientation, memory, and calculation. Living arrangement moderated the association only at the level of internet adoption; specifically, co-residence with children attenuated the protective effect of internet adoption.

**Conclusion:**

Internet use exerts multidimensional and heterogeneous effects on cognitive function among older adults, moderated by living arrangement. These findings provide theoretical guidance for stratified digital intervention design.

## 1 Introduction

Against the backdrop of deepening digitalization and population aging, the impact of internet use on cognitive function among older adults has become a focal point of public health research. As of December 2025, the number of internet users aged 60 and above in China has exceeded 160 million, with an internet penetration rate among older adults reaching 53.7% (1). The internet has thus become an important platform for Chinese older adults to access information, engage in social interactions, and pursue leisure and entertainment (2, 3). Meanwhile, as the country with the largest and fastest-growing older adult population worldwide, China faces unprecedented challenges regarding cognitive function of older adults. According to the China Alzheimer’s Disease Report 2025, the number of individuals with Alzheimer’s disease and related dementias (ADRD) in China reached approximately 16.99 million in 2021, accounting for 29.8% of the global total (4). With the continuing trend of population aging, ADRD cases in China are projected to reach 49.89 million by 2060, with an economic burden exceeding 5 trillion US dollars (5). In this context, whether internet use can serve as a modifiable protective factor to delay cognitive decline among older adults has attracted widespread attention.

A substantial body of research has established the protective effect of internet use on cognitive health in later life (6–9). However, most evidence derives from dichotomous measures of whether used (10–12), reducing complex internet behaviors to a single threshold variable. With the deepening penetration of digital technology, researchers have begun to examine dimensions such as use intensity (13, 14), function types (15, 16), and specific devices (17), yet findings remain inconsistent, diverging in both the direction and magnitude of effects across these dimensions. These inconsistent findings point to the limitations of the binary approach and underscore the value of examining multiple dimensions of internet use to better understand its cognitive effects. Nevertheless, existing studies suffer from three notable limitations in disentangling these differentiated effects. First, most prior research has relied on device type as an indirect indicator of operational complexity, with limited direct examination of operational proficiency. Second, the majority of studies have examined single dimensions in isolation, lacking an integrative comparative framework (18, 19). Third, effect estimates may be biased. Some studies have failed to exclude non-users, confounding the composition of the reference group; others have not controlled for other usage dimensions, obscuring the net effect of specific dimensions (20, 21).

To systematically explain the differentiated effects across these multidimensional dimensions, this study draws upon the technology reserve hypothesis as its core theoretical framework. This hypothesis posits that experiences of technology engagement can build individuals’ technology reserves, which buffer against age-related cognitive decline (22). Its central insight is that the accumulation efficiency of technology reserves is highly contingent upon usage patterns (23). Only operations characterized by high cognitive engagement and goal-directedness can effectively activate neuroplasticity, whereas excessive or passive use is unlikely to yield substantive cognitive protection (24, 25). This hypothesis provides the theoretical foundation for understanding the differentiated cognitive effects of various internet use dimensions.

Within the Chinese familial cultural context, these multidimensional effects are profoundly shaped by living arrangement. Unlike their Western counterparts who prefer living independently, Chinese older adults tend to co-reside with their children. However, influenced by urbanization and changes in family structure, the population of older adults living alone or with only a spouse has been steadily expanding (26). Different living arrangement imply variations in the social resources available to older adults (27). Such differences not only shape their motives and patterns of internet use (28) but may also moderate the pathways through which internet use affects cognitive function. Existing empirical studies suggest that older adults living alone may derive greater cognitive benefits from internet use than those co-residing with children (29). Yet how living arrangement systematically moderates the differentiated effects of various use dimensions remains to be examined.

Grounded in the technology reserve hypothesis, this study examines the impact of internet use on cognitive health among older adults from four dimensions--internet adoption, use intensity, device operation proficiency, and function types--with living arrangement as a moderating variable. Its marginal contributions are twofold. First, it moves beyond the binary measure of internet use by revealing how different usage dimensions differentially affect cognitive function and its subdomains. Second, it demonstrates the moderating role of living arrangement, providing empirical support for residential context–tailored digital interventions.

## 2 Research hypotheses

### 2.1 Internet adoption and cognitive function

Internet adoption constitutes a necessary threshold for the accumulation of technology reserve. Internet adoption exposes older adults to digital information flows and interactive scenarios that inherently involve cognitive challenges such as multitasking, goal orientation, and real-time feedback (22, 23). Research has shown that internet use is significantly associated with older adults’ language ability, attention and calculation skills, orientation, and memory (11, 18). Internet adopters gain cognitive protection through three pathways. First, cognitive stimulation arises from processing multiple information streams when operating digital devices (6), promoting executive function, memory, and problem-solving ability more effectively than traditional cognitive activities (30). Second, social connection is facilitated by video calls and social media, helping older adults maintain contact with family and friends (16, 17) while significantly reducing loneliness and social isolation (31–33)--both of which are established risk factors for cognitive decline (34). Third, digital compensation operates through tools such as smart reminders, GPS navigation, and electronic payment, offsetting memory and executive function decline to support independent daily living (35, 36). Crucially, however, the accumulation of such reserves presupposes actual engagement; internet adoption thus constitutes the necessary threshold condition for this process to occur. Accordingly, this study proposes:

*H*1: Internet adoption is positively associated with cognitive function among older adults.

### 2.2 Multidimensional effects of internet use behavior

The technology reserve hypothesis further posits that the cognitive effects of technology engagement vary across usage patterns (37, 38). Different dimensions of use--including use intensity, device operation proficiency, and functional types--may influence cognitive function through distinct pathways. First, use intensity follows an inverted-U relationship. Moderate and regular use promotes reserve accumulation through sustained cognitive stimulation and social connection (13); longitudinal evidence indicates that higher internet use frequency positively predicts older adults’ episodic memory performance (39). However, both excessive and low-frequency use are unlikely to confer effective protection (21, 40). Second, device operation proficiency exhibits diminishing returns. Higher proficiency facilitates efficient utilization of online resources and activates protective mechanisms; however, continued improvement in proficiency may also be accompanied by increased reliance on devices. Tasks that originally required autonomous cognitive engagement (e.g., memory, calculation, orientation) are gradually outsourced to the devices, reducing the frequency of active cognitive participation and thereby partially offsetting the cognitive protective effects (41). Third, function types diverge based on depth of cognitive engagement. Instrumental use (e.g., online shopping, financial management) involves goal-setting and multi-step operations that constitute intensive cognitive functional training (42, 43), whereas entertainment-oriented use (e.g., short video browsing, passive information reading) primarily entails passive sensory stimulation with limited cognitive engagement depth, failing to generate effective technology reserve accumulation and potentially leading to declines in verbal memory and overall cognitive function (25). Accordingly, this study proposes:

*H*2: Different dimensions of internet use behavior (use intensity, device operation proficiency, and functional types) have differentiated effects on cognitive function among older adults.

### 2.3 The moderating role of living arrangement

Living arrangement constitutes a critical social contextual factor shaping the cognitive effects of internet use. Older adults co-residing with children have access to immediate technical support, skill guidance, and sustained encouragement for use (44), which helps alleviate usage anxiety and maintain regular use patterns (45). However, such intergenerational assistance may also lead to proxy use (46), where older adults rely on family members to complete online tasks, thereby undermining the cognitive protective potential of internet use. By contrast, living alone lack immediate intergenerational technical support, rendering their internet use more autonomous and compensatory (27, 47). They must independently navigate operational challenges and resolve usage problems on their own, a process that precisely activates high-intensity cognitive engagement and may thus yield greater cognitive benefits. Multiple studies have shown that older adults living alone derive greater cognitive benefits from internet use than those co-residing with children (21, 28, 29). Accordingly, this study proposes:

*H*3: Living arrangement moderates the relationship between internet use dimensions and cognitive function among older adults.

## 3 Data and methodology

### 3.1 Data source

**Figure 1:**
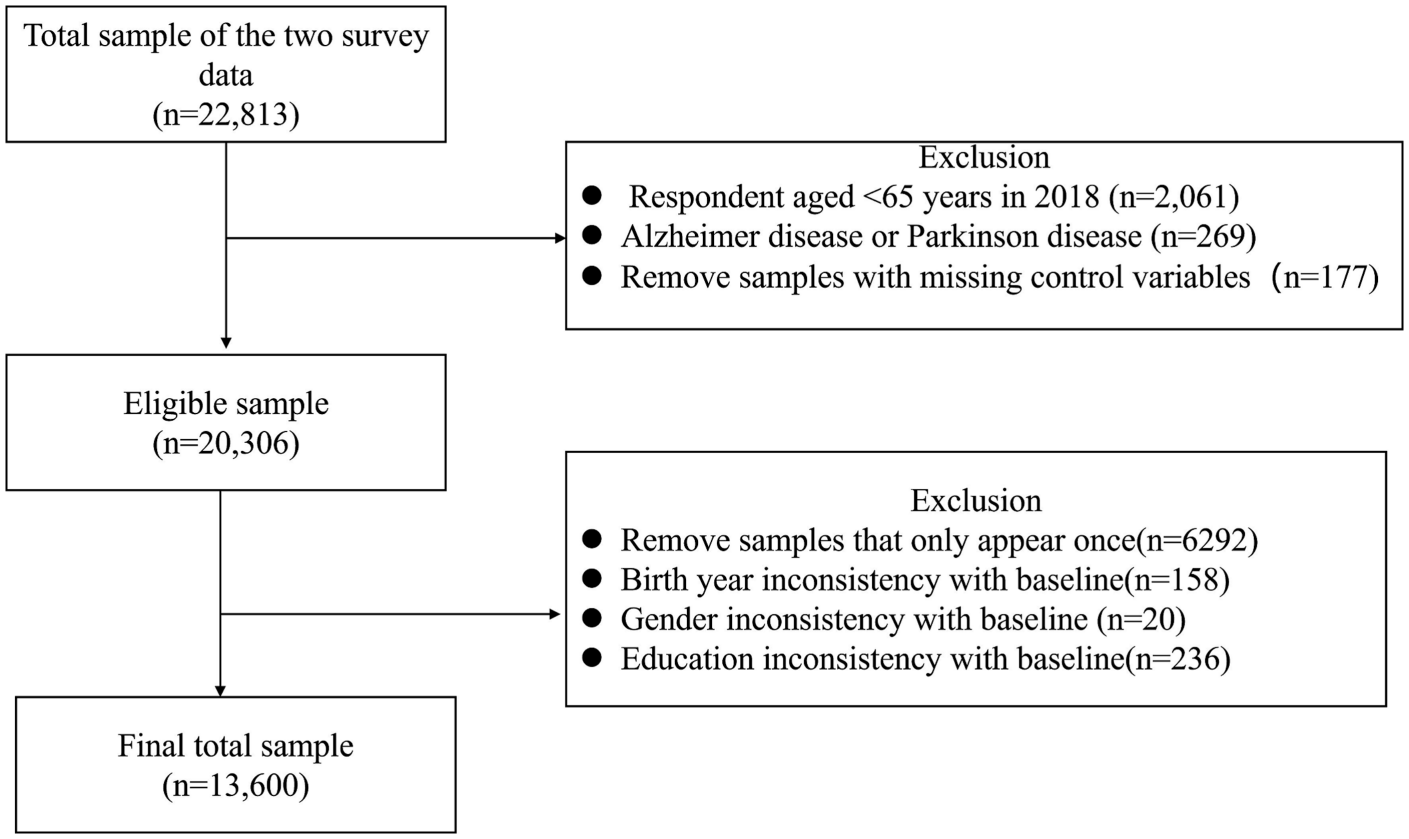
Flowchart of participant selection.

The China Longitudinal Aging Social Survey (CLASS) is a nationally representative longitudinal survey of Chinese adults aged 60 and above. Using stratified multistage probability sampling, CLASS covers 28 provinces in China (excluding Hong Kong, Macao, Taiwan, Hainan, Xinjiang, and Tibet). Four survey waves have been completed, with a baseline survey in 2014 and follow-ups in 2016, 2018, and 2020. CLASS began collecting data on internet use among older adults in 2016, refined the relevant measures in the 2018 wave (e.g., use frequency, operational proficiency), and retained these items in subsequent surveys. To examine the multidimensional effects of internet use while excluding exogenous shocks from the COVID-19 pandemic, this study analyzed the 2018 and 2020 survey waves. The sample selection process is illustrated in Figure 1: (1) respondents aged below 65 at baseline were excluded; (2) those who self-reported a physician-diagnosed dementia or Parkinson’s disease were excluded; (3) observations with missing values in core variables were excluded; (4) respondents who participated in only one wave were excluded; and (5) those with inconsistent demographic characteristics (gender, birth year, or education) across the two waves were excluded. This procedure yielded a balanced panel of 6,800 unique individuals with 13,600 person-wave observations.

### 3.2 Variables

#### 3.2.1 Cognitive function

Cognitive function, the dependent variable, was measured using the Mini-Mental State Examination (MMSE) from the CLASS survey. The MMSE assessed three domains: orientation (temporal, spatial, personal; score range 0–5), memory (immediate/delayed recall; score range 0–6), and calculation (serial subtraction of 7 from 100, repeated five times; score range 0–5). Total scores ranged from 0 to 16, with higher scores indicating better cognitive function. Cronbach’s *α* (0.826 in 2018; 0.824 in 2020) suggested good internal consistency.

#### 3.2.2 Internet use

Internet use is defined as the behavior of older adults accessing the Internet through networked electronic devices (e.g., mobile phones, computers). Drawing on existing research on older adults’ internet use behavior (48, 49), this study operationalizes internet use into the following four dimensions: internet adoption, use intensity, device operation proficiency, and function types.

1. Internet adoption. This binary variable was derived from the CLASS questionnaire item “Do you use the internet?” Respondents who answered “Never use the internet” were coded as 0; all other responses were coded as 1.
2. Use intensity. Measured by frequency of internet use, this variable was constructed also from the same question “Do you use the internet?.” After excluding non-users, “daily use” was coded as 1, while “at least once a week,” “at least once a month,” and “several times a year” were combined and coded as 0.
3. Device operation proficiency. Measured by operational proficiency. This variable was based on the question “How proficient are you in using these devices?” Drawing on path-dependence theory, which posits that older adults tend to access the internet through devices they operate most proficiently (50). This study used the highest proficiency level reported across all internet-enabled devices to represent overall device operation proficiency level. Responses were categorized into two levels: unskilled (“very unskilled,” “somewhat unskilled,” or “moderate” coded as 0), and skilled (“somewhat skilled” or “very skilled,” coded as 1).
4. Function types. Function types were derived from the question “What do you usually do online?” Based on the content of internet activities, 11 response options were consolidated into four categories: (a) social interaction (i.e., voice/video chatting, text chatting); (b) information acquisition (i.e., reading news, browsing non-news articles); (c) entertainment (i.e., listening to music/radio, watching videos, playing games); (d) Instrumental use (i.e., transportation, shopping, education and training, health management, financial investment, education and training, health management). Each category was coded as an independent dummy variable (0 or 1).

#### 3.2.3 Living arrangement

Living arrangement serves as the moderating variable in this study. This variable was constructed based on two questions: “How many people live with you?” and “Who are the people that live and eat with you?” Respondents living with any of the following family members were classified as co-residing with children: daughters, sons, sons-in-law, daughters-in-law, grandchildren and their spouses, or great-grandchildren and their spouses. Co-residence was coded as 1, and non-co-residence was coded as 0.

#### 3.2.4 Control variables

This study statistically controlled for potential confounding variables across four dimensions. First, demographic characteristics included age (continuous variable), gender (male = 1, female = 0), and marital status (married = 1, single/widowed/divorced = 0). Second, health status encompassed the number of chronic conditions and instrumental activities of daily living scores (IADL_S_). The number of chronic conditions was a continuous variable representing the total number of chronic diseases reported by respondents. IADL_S_ were assessed using the Lawton and Brody scale, which examines independent functioning across seven domains: telephone use, shopping, food preparation, housekeeping, transportation, medication management, and financial management. Each item was coded on a three-point scale (1 = no help needed; 2 = some help needed; 3 = completely unable to perform), with total scores ranging from 7 to 21. Third, life satisfaction (very satisfied = 1, somewhat satisfied = 2, moderate = 3, somewhat dissatisfied = 4, and very dissatisfied = 5). Four, socioeconomic status included education (illiterate = 1; primary school or below = 2; middle school = 3; high school or above = 4), residency (rural = 1, urban = 0), and pension insurance (yes = 1, no = 0).

### 3.3 Modeling design

First, this study employs Ordinary Least Squares (OLS) estimation to examine the effect of internet use on cognitive function among older adults. To mitigate potential endogeneity arising from omitted variables, the model controls for both individual fixed effects (μᵢ) and year fixed effects (λt) (Equation 1). The individual fixed effects absorb all time-invariant characteristics (e.g., baseline age, gender, and education), while the year fixed effects capture common temporal shocks. The baseline regression model is specified as follows:

$$\text{cognitiv}e_{\mathrm{it}}=a_{0}+\beta_{1}\text{Interne}t_{\mathrm{it}}+\beta_{2}X_{\mathrm{it}}+\mu_{i}+\lambda_{t}+\varepsilon_{\mathrm{it}}$$ (1)

Where i denotes the individual and t denotes the year. Cognitive_it_ represents the overall cognitive function of individual i in year t, encompassing orientation, memory, and calculation subdomains, with higher scores indicating better cognitive function. Internet_it_ is the key explanatory variable, operationalized as a four-dimensional construct: internet adoption, use intensity, device operation proficiency, and function types. X_it_ represents control variables, including demographic characteristics, life satisfaction, socioeconomic status, and health status. μ_i_ and λ_t_ denote individual fixed effects and year fixed effects, respectively; ε_it_ is the random error term; α_0_ is the intercept; and β_1_ is the parameter of primary interest.

Second, to address potential endogeneity concerns, this study employs instrumental variable (IV) estimation. Following prior research (51), community-level internet adoption rates (the proportion of adopters among other community members) are used as the instrument for individual internet use. This instrument is expected to influence individual adoption through peer effects and local infrastructure, while remaining plausibly exogenous to individual cognitive function. To strengthen the exclusion restriction, community- and city-level covariates that may confound the relationship are controlled for, including local urbanization rate, local education level, community healthcare accessibility, and community social capital (Supplementary Table 1). These controls absorb the direct effects of regional development on cognitive function, allowing community-level adoption to affect cognitive outcomes primarily through individual adoption behavior.

Third, to mitigate selection bias from observable differences between users and non-users, propensity score matching (PSM) with radius matching (caliper = 0.01) is employed, combined with two-way fixed effects.

Fourth, interaction terms between internet use and living arrangement are introduced to examine the moderating role of living arrangement.

## 4 Results

### 4.1 Descriptive statistics

Table 1 presents the baseline characteristics of the sample (*N* = 6,800). Internet users (*n* = 1,049) were significantly younger (*M* = 68.93, SD = 4.10) than non-users (*M* = 72.41, SD = 5.83). Among users, 54.15% (568) were male, 42.99% had completed secondary education, 6.96% were illiterate, and 82.36% (864) were married. Users reported slightly more chronic conditions (*M* = 1.80, SD = 1.62) than non-users (*M* = 1.58, SD = 1.47), but better IADL_S_ (*M*_users_ = 7.31, SD = 1.15 vs. *M*_non-users_ = 7.75, SD = 1.90). Most users did not co-reside with children (73.21%), had pension coverage (86.37%). Users reported higher life satisfaction (*M* = 2.10, SD = 0.79) than non-users (*M* = 2.24, SD = 0.87), with lower scores indicating greater satisfaction. Additionally, 81.12% resided in urban areas. Regarding use patterns among users, 66.35% used the internet daily; 57.67% self-rated as skilled. The three most frequently used functions were social interaction (87.51%), information acquisition (65.59%), and entertainment (52.53%). Notably, users exhibited significantly higher cognitive function (*M* = 14.44, SD = 2.29) than non-users (*M* = 12.76, SD = 3.51). Specifically, users outperformed non-users across overall cognitive dimensions, including orientation (*M*_users_ = 4.73, SD = 0.64 vs. *M*_non-users_ = 4.37, SD = 1.10), memory (*M*_users_ = 5.29, SD = 1.03 vs. *M*_non-users_ = 4.63, SD = 1.53), and calculation (*M*_users_ = 4.42, SD = 1.27 vs. *M*_non-users_ = 3.76, SD = 1.76).

**Table 1:** Baseline characteristics of the sample.

Variables	Internet use (*N* = 6,800)		*p*-value
	No (*n* = 5,751)	Yes (*n* = 1,049)	
Age(years), mean ± SD	72.41 ± 5.83	68.93 ± 4.10	< 0.001
Gender, n (%)			0.024
Female	2,855 (49.64)	481 (45.85)	
Male	2,896 (50.36)	568(54.15)	
Education, n (%)			< 0.001
Illiterate	1790 (31.13)	73 (6.96)	
Primary school or below	2,612 (45.42)	307 (29.08)	
Middle school	1,027 (17.86)	452 (42.99)	
High school or above	322 (5.60)	220 (20.97)	
Marital status, n (%)			< 0.001
Single/divorced/widowed	1885 (32.78)	185 (17.64)	
Married	3,866 (67.22)	864 (82.36)	
Number of chronic diseases, mean ± SD	1.58 ± 1.47	1.80 ± 1.62	< 0.001
IADL_S_, mean ± SD	7.75 ± 1.90	7.31 ± 1.15	< 0.001
Living arrangement, n (%)			< 0.001
Non-co-residence	3,574 (62.15)	768 (73.21)	
Co-residence	2,177 (37.85)	281 (26.79)	
Pension insurance, n (%)			< 0.001
No	1,538 (26.74)	143 (13.63)	
Yes	4,213 (73.26)	906 (86.37)	
Life satisfaction, mean ± SD	2.24 ± 0.87	2.10 ± 0.79	< 0.001
Residence, n (%)			< 0.001
Urban	2,930 (50.95)	851 (81.12)	
Rural	2,821 (49.05)	198 (18.88)	
Use intensity, n (%)			
Non-daily use		353 (33.65)	
Daily use		696 (66.35)	
Device operation proficiency, n (%)			
Unskilled		444 (42.33)	
Skilled		605 (57.67)	
Function types, n (%)			
Social interaction		918 (87.51)	
Entertainment		551 (52.53)	
Information acquisition		688 (65.59)	
Instrumental use		209 (19.92)	
Cognitive function, mean ± SD	12.76 ± 3.51	14.44 ± 2.29	< 0.001
Orientation	4.37 ± 1.09	4.73 ± 0.64	< 0.001
Memory	4.63 ± 1.53	5.29 ± 1.03	< 0.001
Calculation	3.76 ± 1.76	4.42 ± 1.27	< 0.001

### 4.2 Internet adoption and cognitive function

#### 4.2.1 Baseline regression

Table 2 presents baseline regression results examining the association between internet adoption and cognitive function. Model 1, which includes only internet adoption, reveals a statistically significant and positive association between internet adoption and overall cognitive function (*B* = 1.202, *p* < 0.001). Model 2 further adjusts for demographic characteristics, health status, life satisfaction, and socioeconomic factors. Internet adoption remains a significant positive predictor (*B* = 0.842, *p* < 0.001). These findings indicate the cognitive benefits of internet adoption, supporting H1. Regarding control variables, the number of chronic diseases is significantly and positively associated with overall cognitive function (*B* = 0.251, *p* < 0.001), indicating that older adults with more chronic conditions unexpectedly show higher cognitive function. IADL_S_ are significantly and negatively associated with overall cognitive function (*B* = −0.383, *p* < 0.001), suggesting that older adults with poorer functional independence exhibit lower cognitive function. Additionally, older adults with pension insurance (*B* = −0.533, *p* < 0.001) and those with lower life satisfaction (*B* = −0.624, *p* < 0.01) exhibit significantly lower cognitive function.

**Table 2:** Association between internet adoption and cognitive function.

Variables	Model 1	Model 2	Model 3	
	Cognitive function	Cognitive function	Internet adoption	Cognitive function
Internet adoption	1.202^***^ (0.134)	0.842^***^ (0.128)		1.258^***^ (0.342)
Marital status (reference: single/divorced/widowed)				
Married		0.144 (0.162)	−0.012 (0.011)	0.162 (0.159)
Number of chronic diseases		0.251^***^ (0.041)	−0.000 (0.004)	0.251^***^ (0.041)
IADL_S_		−0.383^***^ (0.032)	−0.012^***^ (0.002)	−0.384^***^ (0.032)
Living arrangement (reference: non-co-residence)				
Co-residence		−0.236 (0.123)	−0.041^**^ (0.014)	−0.240 (0.126)
Pension insurance (reference: no)				
Yes		−0.533^***^ (0.163)	0.009 (0.013)	−0.439^**^ (0.165)
Life satisfaction		−0.624^***^ (0.065)	−0.008 (0.007)	−0.615^***^ (0.065)
Residence (reference: urban)				
Rural		0.053 (0.192)	−0.006 (0.018)	0.028 (0.193)
Community-level internet adoption rates			0.778^***^ (0.037)	
Local urbanization rate			−0.054 (0.046)	1.464^***^ (0.428)
Local education level			0.032 (0.071)	−3.536^***^ (0.782)
Community healthcare accessibility			−0.021^*^ (0.009)	−0.228^*^ (0.089)
Community social capital			−0.036 (0.033)	−0.598 (0.383)
Individual fixed effects	Yes	Yes	Yes	Yes
Year fixed effect	Yes	Yes	Yes	Yes
Cragg-Donald Wald F				2966.589
Observations	13,600	13,600	13,600	13,600

^*^p < 0.05, ^**^p < 0.01, ^***^p < 0.001. Standard errors are reported in parentheses.

#### 4.2.2 Endogeneity and robustness checks

1. Instrumental variable estimates. As described in the methods section, the community-level internet adoption rate is used as an instrument for individual internet use. The first-stage regression shows a strong positive association between community-level internet adoption prevalence and individual internet adoption (*B* = 0.778, *p* < 0.001). The Cragg-Donald Wald F-statistic (2966.59) far exceedes the Stock-Yogo critical value, rejecting the null hypothesis of weak instrumentation (Model 3, Table 2). The second-stage results show that internet adoption remains significantly and positively associated with overall cognitive function (*B* = 1.258, *p* < 0.001, Model 3, Table 2), consistent with the baseline estimates. The IV estimate (*B* = 1.258) exceeds the OLS estimate (*B* = 0.842), a pattern consistent with prior studies using similar instruments (52). This result supports a local average treatment effect (LATE) interpretation (53): the IV estimator captures the effect for compliers--those induced to adopt internet use by higher community penetration--whose cognitive gains from initial adoption may exceed the average treatment effect (ATE) in the full population.
2. Propensity score matching estimates. As an additional check on selection bias, radius matching (caliper = 0.01) combined with two-way fixed effects is employed. The matching successfully balances all covariates, as indicated by standardized differences below 10% after matching (Supplementary Table 2). The PSM estimate based on the matched sample (*N* = 13,390) remains consistent with the primary findings (*B* = 0.849, *p* < 0.001; Supplementary Table 3).
3. Selective attrition. Given the longitudinal design, retention rates are further examined by baseline internet use status. Non-users have an attrition rate of 24.66% (1,882/7,633), compared to 17.27% (219/1,268) for users, a statistically significant difference (χ^2^ = 32.88, *p* < 0.001; Supplementary Table 4). This pattern is consistent with findings from other longitudinal aging studies, in which individuals with lower cognitive resources and weaker social participation are more likely to drop out of follow-up surveys (54). Although selective retention may bias the estimated internet effects toward individuals with higher cognitive reserve, this concern does not undermine the main conclusions. First, the PSM estimates (*B* = 0.849) are consistent with the OLS results (*B* = 0.842), suggesting that observable selectivity, including factors related to attrition, does not drive the findings. Second, the IV estimates support the positive association and are less susceptible to sample composition shifts because they identify complier effects. These compliers are likely to include individuals with lower baseline cognitive reserve. Taken together, the protective association between internet adoption and cognitive function proves robust across different estimation strategies and sample specifications.

#### 4.2.3 Heterogeneous effects on cognitive subdomains

Cognitive function is multidimensional; thus, a composite score may obscure domain-specific effects. This study therefore analyzes three core domains--orientation, calculation, and memory--as separate outcomes. Model 4 in Table 3 presents these results. Columns (1) to (3) show that internet adoption exerts significant positive effects on orientation (*B* = 0.406, *p* < 0.001), calculation (*B* = 0.416, *p* < 0.01), and memory (*B* = 0.437, *p* < 0.05). These domain-specific results align with the main findings using the composite score, demonstrating that the cognitive benefits of internet adoption extend across core cognitive domains.

**Table 3:** Heterogeneous effects on cognitive domains.

Variables	Model 4		
	(1) Orientation	(2) Memory	(3) Calculation
Internet adoption	0.406^***^(0.100)	0.416^**^(0149)	0.437^*^(0.192)
Control variables	Yes	Yes	Yes
Individual fixed effects	Yes	Yes	Yes
Year fixed effect	Yes	Yes	Yes
Observations	13,600	13,600	13,600

^*^p < 0.05, ^**^p < 0.01, ^***^p < 0.001. Standard errors are reported in parentheses.

### 4.3 Use intensity, device operation proficiency, function types, and cognitive function

To further investigate the associations between specific patterns of internet use and cognitive function among older adults, this study restricted the analytical sample to respondents who used the internet in both waves (*n* = 1,634; Supplementary Table 5). Model 5 jointly controls for all core independent variables. Variance inflation factor (VIF) analysis indicates that all VIF values are below 10, with a mean VIF of 1.15, suggesting that severe multicollinearity is not a concern. After controlling for other variables, use intensity exerts a significant positive effect on overall cognitive function (*B* = 0.587, *p* < 0.05), indicating a protective effect of daily internet use on overall cognitive function among older adults. However, device operation proficiency is significantly and negatively associated with overall cognitive function (*B* = −0.504, *p* < 0.05); that is, older adults categorized as skilled exhibit lower cognitive scores than unskilled counterparts. Regarding function types, only instrumental use (e.g., online shopping, financial services) demonstrates a significant positive effect on overall cognitive function (*B* = 0.497, *p* < 0.05), whereas social interaction, entertainment, and information acquisition show no statistically significant associations. Model 6 further examines the effects of each dimension on specific cognitive domains. The results show that use intensity significantly improves orientation (*B* = 0.298, *p* < 0.01). Device operation proficiency is significantly and negatively associated with memory (*B* = −0.271, *p* < 0.05). Instrumental use significantly enhances memory (*B* = 0.396, *p* < 0.01). For the calculation subdomain, the associations with use intensity, device operation proficiency, and functional types are not statistically significant. Collectively, these findings indicate that different dimensions of internet use exert heterogeneous effects on cognitive function among older adults, supporting H2 (Table 4).

**Table 4:** Relationship between use intensity, device operation proficiency, function types, and cognitive function.

Variables	Model 5		Model 6					
			(1) Orientation		(2) Memory		(3) Calculation	
	B	SE	B	SE	B	SE	B	SE
Use intensity (reference: non-daily use)								
Daily use	0.587^*^	0.275	0.298^***^	0.071	0.067	0.136	0.223	0.171
Device operation proficiency (reference: unskilled)								
Skilled	−0.504^*^	0.224	0.007	0.069	−0.271^*^	0.112	−0.240	0.133
Function types								
Social interaction	−0.218	1.044	0.235	0.329	−0.070	0.400	−0.383	0.616
Entertainment	0.135	0.206	0.003	0.041	0.167	0.104	−0.035	0.115
Information acquisition	0.203	0.226	0.006	0.059	0.130	0.107	0.067	0.151
Instrumental use	0.497^*^	0.208	−0.111	0.067	0.396^***^	0.108	0.212	0.125
Control variables	Yes		Yes		Yes		Yes	
Individual fixed effects	Yes		Yes		Yes		Yes	
Year fixed effect	Yes		Yes		Yes		Yes	
R-squared	0.919		0.942		0.880		0.894	
Observations	1,634		1,634		1,634		1,634	

^*^p < 0.05, ^**^p < 0.01, ^***^p < 0.001.

### 4.4 Moderating role of living arrangement

Table 5 reports the moderating effects of living arrangement on various dimensions of internet use. Model 7 reveals a significant negative interaction between internet adoption and living arrangement (*B* = −0.524, *p* < 0.05), indicating that co-residence with children attenuates the positive effect of internet adoption on overall cognitive function. That is, older adults living with children derive relatively limited cognitive benefits from internet adoption. To further elucidate the moderating mechanisms, this study restricts the analytical sample to respondents who used the internet in both waves (*n* = 1,634) and examines how living arrangement moderates the associations between specific internet use behaviors and overall cognitive function in Model 8. The results indicate that the moderating effects of living arrangement on use intensity, device operation proficiency and function types fail to reach statistical significance. In summary, the moderating effect of living arrangements is only significant at the internet adoption level. Co-residence with children attenuates the cognitive benefits of internet adoption, providing partial support for H3.

**Table 5:** The moderating role of living arrangement in the association between internet use and cognitive function.

Variables	Model 7		Model 8	
	B	SE	B	SE
Internet adoption	1.024^***^	0.141		
Living arrangement	−0.119	0.143	1.768	1.607
Internet adoption × Living arrangement	−0.524^*^	0.237		
Use intensity			0.427	0.339
Use intensity × Living arrangement			0.335	0.566
Device operation proficiency			−0.226	0.265
Device operation proficiency × Living arrangement			−0.678	0.508
Function types				
Social interaction			1.152	1.502
Social interaction × Living arrangement			−2.799	1.658
Entertainment			0.007	0.222
Entertainment × Living arrangement			0.346	0.427
Information acquisition			0.151	0.240
Information acquisition × Living arrangement			0.202	0.487
Instrumental use			0.350	0.241
Instrumental use × Living arrangement			0.583	0.487
Control variables	Yes		Yes	
Individual fixed effects	Yes		Yes	
Year fixed effect	Yes		Yes	
R-squared	0.836		0.924	
Observations	13,600		1,634	

Robust standard errors are in parentheses. ^*^p < 0.05, ^**^p < 0.01, ^***^p < 0.001.

## 5 Discussion

This study uses two-wave data from the China Longitudinal Aging Social Survey (CLASS) to examine the relationship between internet use and cognitive function among older adults. First, internet adoption demonstrates a protective effect on cognitive function. Second, among sustained internet users, different dimensions of use exhibit differential associations: use intensity and instrumental use are positively associated with cognitive function, whereas device operation proficiency is negatively associated with cognitive function. Further analyses reveal significant heterogeneity in how these dimensions affect distinct cognitive domains, including orientation, memory, and calculation. In addition, the moderating role of living arrangement is significant only at the internet adoption level, with co-residence with children attenuating the protective effect of internet adoption on cognitive function.

### 5.1 Cognitive protective effects of internet adoption

Internet adoption exerts a significant positive effect on cognitive function, aligning with prior research (55). Drawing on the technology reserve hypothesis, this protective effect may operate through multiple pathways, including multidimensional cognitive stimulation afforded by internet use (43, 56), enhanced social connections (57, 58), and digital compensation mechanisms (10). Nevertheless, given that these potential mediating pathways were not directly tested in the present study, further research is required to clarify the underlying mechanisms.

### 5.2 Differential impacts of internet use behaviors

#### 5.2.1 Positive effects of use intensity

The positive association between use intensity and cognitive function is supported in this study, consistent with prior research indicating that higher frequency of internet use is associated with greater cognitive benefits (29). From the perspective of the technology reserve hypothesis, this association reflects the cumulative effect of sustained technology engagement. Regular internet use imposes a stable cognitive load on the brain, thereby helping maintain processing speed and executive function efficiency (39, 56). However, the measure of use intensity in the present study did not distinguish between moderate and excessive use. Existing longitudinal research suggests that internet use exhibits an inverted U-shaped relationship with health outcomes in later life (59). Excessive internet immersion may lead to attention fragmentation (60), sleep disruption (61), and reduced face-to-face social interaction (62) which could adversely affect cognitive function over time. Therefore, these findings should not be interpreted simplistically as “more is always better,” but rather suggest that moderate and regular patterns of use may be more advisable.

#### 5.2.2 Negative effects of device operation proficiency

A noteworthy finding is that device operation proficiency is negatively associated with cognitive function. This result appears to contradict studies emphasizing the benefits of digital literacy (17, 24, 28), but can be understood from the following perspectives. First, measurement validity concerns. Proficiency in this study was measured through self-reports by older adults, which may introduce measurement bias. Self-reported proficient users could be genuinely skilled individuals, or they could be heavy users who engage only in simple, repetitive operations. Therefore, interpretations of this finding should be treated with caution. Second, reverse causality and unobserved confounding cannot be ruled out. Older adults with poorer cognitive function may engage more frequently in simple, repetitive internet activities, thereby developing higher proficiency through repeated practice, while their cognitive level itself remains low. Additionally, unobserved confounders (e.g., usage motivation) may also exist. Third, cognitive offloading effects. If self-reported proficiency does reflect higher digital skills, proficient users may delegate cognitively demanding tasks (e.g., memory, mental arithmetic, and spatial navigation) to digital devices, potentially leading to “disuse atrophy” of these abilities (63). Future research should validate this using objective measures such as task-based performance tests.

#### 5.2.3 Protective effects of instrumental use

Among various types of internet functions, only instrumental use is significantly associated with improved cognitive function. This finding is consistent with prior research (42). The protective effect may stem from the inherent task complexity of instrumental activities. Activities such as online shopping, and financial management involve goal-directed problem-solving, multi-step reasoning, and numerical information processing, which promote deeper cognitive engagement and sustained attention, thereby providing effective cognitive stimulation. This aligns with the technology reserve hypothesis, which posits that engagement in cognitively challenging activities helps enhance cognitive reserve in the aging brain (43). By contrast, social interaction, entertainment and information acquisition are not significantly associated with cognitive function. This finding appears to diverge from some previous studies (19), but can be explained from two perspectives. First, most prior studies did not simultaneously include all types of internet activities in the same model, nor did they exclude non-users from the analytical sample, both of which may have introduced estimation bias. Second, from a cognitive engagement perspective, social interaction, entertainment, and information acquisition activities tend to rely more on shallow processing (64), with users often in a passive information-receiving state (25), resulting in lower cognitive engagement and limited independent cognitive benefits. These findings suggest that cognitive protective effects are not a universal consequence of function types, but rather depend on the cognitive complexity of specific functions.

### 5.3 Heterogeneous effects of internet use dimensions across cognitive subdomain

This study finds that internet adoption exerts an overall beneficial effect on cognitive subdomains among older adults, specifically manifesting as significant improvements in orientation, memory, and calculation. This finding is consistent with prior evidence (11, 18). Further analyses reveal heterogeneous effects of different dimensions of internet use across cognitive subdomains. Regarding orientation, a substantial body of research indicates that internet use is associated with better orientation (18, 65), and that sustained users outperform those who discontinue use (13). Daily internet use provides stable and regular stimulation, requiring older adults to continuously process temporal, spatial, and contextual information. This routine input of multi-source spatiotemporal information strengthens their perception and integration of time flow, spatial location, and current environment. Regarding memory, instrumental use significantly enhances memory, whereas device operation proficiency is negatively associated with memory. As a cognitively challenging technological activity, instrumental use itself constitutes an effective exercise for the memory system, engaging working memory, episodic memory, and prospective memory. This multidimensional memory engagement aligns with the neuroplasticity principle of “use it or lose it.” Longitudinal analysis confirms that initiating online medication refill services is significantly associated with improved episodic memory (66). Notably, the negative association between device operation proficiency and memory may stem from proficient users’ tendency to rely on external digital tools as substitutes for internal memory, resulting in a cognitive offloading effect; it may also be attributable to measurement error and reverse causality, warranting cautious interpretation. These findings suggest that the cognitive protective effects of internet use are not uniformly distributed across cognitive subdomains, which has direct implications for the design of precision digital interventions.

### 5.4 Moderating effects of living arrangement on internet use and cognitive function

Moderation analyses reveal that the moderating effect of living arrangement is significant only at the level of internet adoption. Specifically, older adults living alone derive greater cognitive benefits from internet use than those residing with children, whereas no significant moderating effects are observed for use intensity, device operation proficiency, or function types. This finding carries two implications. First, living arrangement primarily influences the decision of whether to adopt the internet, rather than the behavioral patterns of how to use it. Once older adults have crossed the adoption threshold, the moderating role of living arrangement no longer operates. Second, the greater cognitive benefits observed among older adults living alone are consistent with prior research and support the compensation mechanism (67). Internet use compensates for the structural deficit in offline social interaction and cognitive stimulation among older adults living alone, thereby generating higher marginal cognitive returns (68). The attenuated cognitive benefits among those residing with children may be partly attributable to the substitution effect of intergenerational support on autonomous use. Although intergenerational digital support improves internet accessibility and convenience (69) reliance on family members to complete online tasks may weaken the cognitive protective effects. These findings suggest that digital intervention strategies should be designed with full consideration of older adults’ living arrangement.

### 5.5 Limitations

The present study has several limitations. First, although fixed-effects estimation accounts for time-invariant omitted variable bias, it cannot rule out potential confounding from time-varying unobserved factors. Future research could further address time-varying confounding through more intensive longitudinal measurements and quasi-experimental designs. Second, data limitations preclude direct comparisons of cognitive outcomes between attritors and stayers, despite various robustness checks. Future studies with improved retention strategies for disadvantaged groups would help clarify the cognitive effects of internet use. Third, the mediating mechanisms posited by the technology reserve hypothesis, including cognitive stimulation, social connection, and digital compensation, have not been directly tested. Future mediation analyses are needed to identify the pathways linking internet use to cognitive function. Fourth, the absence of usage duration indicators precludes the examination of potential dose–response effects. Future research should adopt more refined measures of usage duration to fill this gap. Fifth, device operation proficiency was assessed using self-reported measures with limited precision. Future studies employing objective performance-based measures could help clarify whether the negative association between proficiency and cognitive function reflects technology dependence that substitutes for intrinsic cognitive effort.

## 6 Conclusion

Drawing on the technology reserve hypothesis and two-wave panel data from the China Longitudinal Aging Social Survey (CLASS), this study systematically examined the associations between multidimensional internet use and cognitive function among older adults, as well as the moderating role of living arrangement. Three main findings emerged. First, internet adoption exerts protective effects on both global cognitive function and specific subdomains, including orientation, memory, and calculation. Second, among sustained users, use intensity and instrumental use facilitate cognitive improvement, whereas device operation proficiency is negatively associated with cognitive function, with these effects exhibiting heterogeneity across cognitive subdomains such as orientation and memory. Third, the moderating effect of living arrangement is significant only at the level of internet adoption, with co-residence with children attenuating the cognitive protective effects of internet adoption.

These findings carry clear practical implications. First, digital intervention strategies should be oriented toward high-quality use, encouraging older adults to maintain regular usage habits and actively expand instrumental use scenarios, while remaining vigilant against the risks of excessive use. Second, interventions should be tailored to specific cognitive domains. For maintaining orientation, daily use is recommended to reinforce spatiotemporal information processing; for improving memory, multi-step, goal-directed instrumental tasks should be emphasized. Third, living arrangement should serve as a stratifying factor in intervention design. For older adults living alone, interventions should focus on integrating internet use into daily life to establish stable sources of cognitive stimulation. For those residing with adult children, autonomous digital engagement should be encouraged, in order to enhance cognitive participation.

## Data Availability

Publicly available datasets were analyzed in this study. This data can be found at: the data are publicly available through the CLASS official website: http://class.ruc.edu.cn/.

## References

[1] China Internet Network Information Center. The 57th Statistical Report on China’s Internet Development (2026). Available online at: https://www.cnnic.net.cn/n4/2026/0304/c88-11549.html (Accessed April 26, 2026)

[2] Huang J Ge Z Chu Y Yan Y Zhang W Liang H . Associations between social media use and anxiety and depression among older adults: cross-sectional study. JMIR Aging. (2025) 8:e71712. doi: 10.2196/71712, 41216814 PMC12603664

[3] Li M Lin Z Li Y. Rethinking eldercare in a digital age: internet use and shifting attitudes in China. Innov Aging. (2025) 9:igaf122.4358. doi: 10.1093/geroni/igaf122.4358

[4] Zhi N Ren R Qi J Liu X Yun Z Lin S . The China Alzheimer report 2025. Gen Psychiatr. (2025) 38:e102020. doi: 10.1136/gpsych-2024-102020, 40792123 PMC12336476

[5] Liu X Chen S Zhang D Gu Y Li G Wu B . Projected prevalence and economic burden of Alzheimer’s disease and related dementias in China: regional disparities and policy implications. Health Data Sci. (2025) 5:0377. doi: 10.34133/hds.0377, 41278023 PMC12635023

[6] Chae HJ Lee SH. Effectiveness of online-based cognitive intervention in community-dwelling older adults with cognitive dysfunction: a systematic review and meta-analysis. Int J Geriatr Psychiatry. (2023) 38:e5853. doi: 10.1002/gps.5853, 36468299 PMC10107881

[7] Cruvinel AC Naves RP Cruvinel NV Guerra AJS Cruvinel RT Melo MFO . Impacts of Digital Technologies on the Cognitive Performance of Older Adults: A Systematic Literature Review Proceedings, 137:24. doi: 10.3390/proceedings2026137024

[8] Gavelin HM Lampit A Hallock H Sabatés J Bahar-Fuchs A. Cognition-oriented treatments for older adults: a systematic overview of systematic reviews. Neuropsychol Rev. (2020) 30:167–93. doi: 10.1007/s11065-020-09434-8, 32266520 PMC7305099

[9] Yuan T Liu K Liang L Zheng C Cui Y Li H . Associations between internet use, social participation, and cognitive function among middle-aged and older adults. npj Mental Health Res. (2025) 4:47. doi: 10.1038/s44184-025-00162-6, 41028880 PMC12485158

[10] Berner J Comijs H Elmståhl S Welmer A-K Sanmartin Berglund J Anderberg P . Maintaining cognitive function with internet use: a two-country, six-year longitudinal study. Int Psychogeriatr. (2019) 31:929–36. doi: 10.1017/S1041610219000668

[11] Liu J Wang Y Wu Q Liu X Hu B. A study on the relationship between internet use and cognitive functioning of older adults under the perspective of smart aging. Front Public Health. (2025) 13:1546929. doi: 10.3389/fpubh.2025.1546929, 40692886 PMC12277142

[12] Quialheiro A Figueiró TH Rech CR Marques LP Paiva KMD Xavier AJ . Can internet use reduce the incidence of cognitive impairment? Analysis of the EpiFloripa aging cohort study (2009–2019). Prev Med. (2022) 154:106904. doi: 10.1016/j.ypmed.2021.106904 34863810

[13] Chen B Yang C Ren S Li P Zhao J. Relationship between internet use and cognitive function among middle-aged and older Chinese adults: 5-year longitudinal study. J Med Internet Res. (2024) 26:e57301. doi: 10.2196/57301, 39539034 PMC11660964

[14] Cho H Choi M Lee H. Mobile internet use and life satisfaction among older adults: the moderating effect of living alone. J Appl Gerontol. (2024) 43:841–9. doi: 10.1177/07334648231216383, 37982396

[15] Jeong HS Lee YW Rhee TG Shim SR. Efficacy of digital therapeutic applications for cognitive training among older adults with mild cognitive impairment or dementia: a systematic review and network meta-analysis of randomized controlled trials. Psychiatry Res. (2025) 348:116426. doi: 10.1016/j.psychres.2025.116426, 40073511

[16] Pan Y Liu Z. The impact of internet usage on cognitive impairment among Chinese older people: a social participation perspective. BMC Geriatr. (2025) 26:198. doi: 10.1186/s12877-025-06846-0, 41372826 PMC12895978

[17] Wang J Zhang N Huang C Wu Q Tong J. Internet use, physical activity, and cognitive function in Chinese older adults: a cross-lagged panel analysis. Front Aging Neurosci. (2025) 17:1579874. doi: 10.3389/fnagi.2025.1579874, 40405917 PMC12095236

[18] Fan Y Wei H Tao Q. Multidimensional internet use related to cognitive performance in older persons: a nationwide cross-sectional study. Front Public Health. (2024) 12:1492331. doi: 10.3389/fpubh.2024.1492331, 39737460 PMC11683067

[19] Yu Y Lv J Liu J Chen Y Chen K Yang Y. Association between living arrangements and cognitive decline in older adults: a nationally representative longitudinal study in China. BMC Geriatr. (2022) 22:843. doi: 10.1186/s12877-022-03473-x, 36348275 PMC9644618

[20] Chen H He M Xu X Atkin D. Examining older adults’ vulnerability to online health scams: insights from routine activity theory. Front Public Health. (2025) 13:1585851. doi: 10.3389/fpubh.2025.1585851, 40371286 PMC12074955

[21] Cho G Betensky RA Chang VW. Internet usage and the prospective risk of dementia: a population-based cohort study. J American Geriatrics Society. (2023) 71:2419–29. doi: 10.1111/jgs.18394, 37132331 PMC13105409

[22] Benge JF Scullin MK. Implications for technological reserve development in advancing age, cognitive impairment, and dementia. Behav Brain Sci. (2020) 43:e157. doi: 10.1017/S0140525X20000126, 32772985 PMC12323679

[23] Benge JF Scullin MK. A meta-analysis of technology use and cognitive aging. Nat Hum Behav. (2025) 9:1405–19. doi: 10.1038/s41562-025-02159-9, 40229575 PMC12333551

[24] Mu A Yuan S Liu Z. Internet use and depressive symptoms among Chinese older adults: two sides of internet use. Front Public Health. (2023) 11:1149872. doi: 10.3389/fpubh.2023.1149872, 36969621 PMC10034360

[25] Shaleha R Roque N. From screens to cognition: a scoping review of the impact of screen time on cognitive function in midlife and older adults. Digit Health. (2025) 11:20552076251343989. doi: 10.1177/20552076251343989, 40656848 PMC12254657

[26] Zhou Z Mao F Ma J Hao S Qian ZM Elder K . A longitudinal analysis of the association between living arrangements and health among older adults in China. Res Aging. (2018) 40:72–97. doi: 10.1177/0164027516680854 27932626

[27] Zhong R Ning W. Impact of living arrangements and internet use on the mental health of Chinese older adults. Front Public Health. (2024) 12:1395181. doi: 10.3389/fpubh.2024.1395181, 39712316 PMC11659149

[28] Yan Y Xing H. Technology for sustainable living: the impact of digital inclusion on the health of China’s elderly living alone. SSM Popul Health. (2025) 29:101751. doi: 10.1016/j.ssmph.2025.101751, 39886258 PMC11780947

[29] Li Y Han W Hu M. Does internet access make a difference for older adults’ cognition in urban China? The moderating role of living arrangements. Health Social Care Comm. (2022) 30:e909–20. doi: 10.1111/hsc.13493, 34245201

[30] Shin SH Park S Wright C D’astous VA Kim G. The role of polygenic score and cognitive activity in cognitive functioning among older adults. Gerontologist. (2021) 61:319–29. doi: 10.1093/geront/gnaa073 32564085

[31] Benge JF Aguirre A Scullin MK Kiselica AM Hilsabeck RC Paydarfar D . Internet-enabled Behaviors in older adults during the pandemic: patterns of use, psychosocial impacts, and plans for continued utilization. Work Aging Retire. (2024) 10:6–13. doi: 10.1093/workar/waac026, 38196827 PMC10772966

[32] Klun M Dosedel T Seljak P Grintal B Kavčič ZM Goriup J . How internet use relates to mental health in older adults: findings from the Czech Republic and the Republic of Slovenia. Front Public Health. (2025) 13:1679931. doi: 10.3389/fpubh.2025.1679931, 41383294 PMC12689409

[33] Xie L Yang H Lin X Ti S Wu Y Zhang S . Does the internet use improve the mental health of Chinese older adults? Front Public Health. (2021) 9:673368. doi: 10.3389/fpubh.2021.673368, 34336769 PMC8322678

[34] Lara E Caballero FF Rico-Uribe LA Olaya B Haro JM Ayuso-Mateos JL . Are loneliness and social isolation associated with cognitive decline? Int J Geriatr Psychiatry. (2019) 34:1613–22. doi: 10.1002/gps.5174, 31304639

[35] Kiselica AM Hermann GE Scullin MK Benge JF. Technology that CARES: enhancing dementia care through everyday technologies. Alzheimers Dement. (2024) 20:8969–78. doi: 10.1002/alz.14192, 39508340 PMC11667529

[36] Scullin MK Jones WE Phenis R Beevers S Rosen S Dinh K . Using smartphone technology to improve prospective memory functioning: a randomized controlled trial. J Am Geriatr Soc. (2022) 70:459–69. doi: 10.1111/jgs.17551, 34786698 PMC8821124

[37] Resor J Cooke S Katz B. The role of social communication technologies in cognition and affect in older adults. Ageing Soc. (2023) 43:24–52. doi: 10.1017/S0144686X21000386

[38] Zhang C Wang Y Wang J Liu X. Does internet use promote mental health among middle-aged and older adults in China? Front Psychol. (2022) 13:999498. doi: 10.3389/fpsyg.2022.999498, 36457930 PMC9706203

[39] Toh WX Khoo SS. Longitudinal associations of internet use with cognitive and social resources among older adults. Comput Hum Behav Rep. (2025) 17:100598. doi: 10.1016/j.chbr.2025.100598

[40] Yu X Mu A Wu X Zhou L. Impact of internet use on cognitive decline in middle-aged and older adults in China: longitudinal observational study. J Med Internet Res. (2022) 24:e25760. doi: 10.2196/25760, 35072642 PMC8822429

[41] Wang D Liu X Chen K Gu C Zhao H Zhang Y . Risks and protection: a qualitative study on the factors for internet addiction among elderly residents in Southwest China communities. BMC Public Health. (2024) 24:531. doi: 10.1186/s12889-024-17980-6, 38378524 PMC10880227

[42] Choi K Ko Y. The relationship between social frailty and cognitive impairment among older adults: the role of various types of internet use. Front Public Health. (2024) 12:1424465. doi: 10.3389/fpubh.2024.1424465, 39310909 PMC11412848

[43] Wang K Chen XS Kang S-Y Smith BD Gu D. Older adults’ online activities and cognition: investigating the psychological mechanisms and age and gender differences. Soc Sci Med. (2024) 352:116988. doi: 10.1016/j.socscimed.2024.116988, 38820692

[44] Liu Z Zadeh RS. Connecting generations: an integrative review of intergenerational technology programs and older adults’ technology use. Gerontol Geriatr Educ. (2025) 46:603–25. doi: 10.1080/02701960.2025.2551963, 40856201

[45] Zhou J Zhao Q Zhou J. Smart senior care cognition and health among Chinese elderly: a moderated mediation model featuring parent-child relationship and internet use. Curr Psychol. (2024) 43:4374–86. doi: 10.1007/s12144-023-04555-8, 37359618 PMC10116446

[46] Grošelj D Reisdorf BC Petrovčič A. Obtaining indirect internet access: an examination how reasons for internet non-use relate to proxy internet use. Telecommun Policy. (2019) 43:213–24. doi: 10.1016/j.telpol.2018.07.004

[47] Furlan C Meggiolaro S. Life satisfaction and internet use in later life in Italy: the role of online activities and living arrangements. Soc Indic Res. (2025) 177:1007–25. doi: 10.1007/s11205-025-03546-5

[48] Petrovcic A Boot WR Burnik T Dolnicar V. Improving the measurement of older adults’ mobile device proficiency: results and implications from a study of older adult smartphone users. IEEE Access. (2019) 7:150412–22. doi: 10.1109/ACCESS.2019.2947765

[49] Xia X Zhu M. The impact of digital literacy on older adults’ utilization of community-based home care services: a cross-sectional study. BMC Health Serv Res. (2025) 25:1367. doi: 10.1186/s12913-025-13427-9, 41094488 PMC12522777

[50] Yue Y Langjie H Yuen LC Wang FH. Bridging the older adult digital divide through the digital habitus. J Aging Res. (2026) 2026:6656355. doi: 10.1155/jare/6656355 41550601 PMC12811827

[51] Xiang J Xing H. The promotion mechanism of physical and mental health of the elderly in China: the impact of the digital divide and social capital. BMC Public Health. (2025) 25:2457. doi: 10.1186/s12889-025-23411-x, 40660167 PMC12261863

[52] Xia X Yuan P Zhao X Jia FR Li B Cai P. Effect of internet use on cognitive function of middle-aged and elderly adults in China: evidence from China family panel studies. Journal of Alzheimer’s Disease Reports. (2024) 8:387–97. doi: 10.3233/ADR-230137, 38549635 PMC10977455

[53] Angrist JD Imbens GW Average Causal Response with Variable Treatment Intensity. NBER Working Paper (1995) t0127. doi: 10.3386/t0127

[54] Jacobsen E Ran X Liu A Chang C-CH Ganguli M. Predictors of attrition in a longitudinal population-based study of aging. Int Psychogeriatr. (2021) 33:767–78. doi: 10.1017/S1041610220000447, 32301414 PMC7572515

[55] Wolters FJ Chibnik LB Waziry R Anderson R Berr C Beiser A . Twenty-seven-year time trends in dementia incidence in Europe and the United States: the Alzheimer cohorts consortium. Neurology. (2020) 95:e519–31. doi: 10.1212/WNL.0000000000010022, 32611641 PMC7455342

[56] Ordonez TN Yassuda MS Cachioni M. Elderly online: effects of a digital inclusion program in cognitive performance. Arch Gerontol Geriatr. (2011) 53:216–9. doi: 10.1016/j.archger.2010.11.007 21131070

[57] Deng Y Liu L Yang Q Yu T. The impact of internet use on cognitive function in older adults: based on the cognitive reserve hypothesis. J Health Psychol. (2025) 31:2110–27. doi: 10.1177/13591053251371788, 41055540

[58] Xiang S Deng Q Chen B. Relationship between internet use and offline leisure activities among Chinese older adult people: a moderated mediation model. Front Public Health. (2025) 12:1458413. doi: 10.3389/fpubh.2024.1458413, 39845655 PMC11753352

[59] Li Y Cai J Yu H. The double-edged sword of internet use in China’s aging population: thresholds, mediation and digital health policy. Front Public Health. (2025) 13:1643510. doi: 10.3389/fpubh.2025.1643510, 40740355 PMC12307157

[60] Firth J Torous J Stubbs B Firth JA Steiner GZ Smith L . The “online brain”: how the internet may be changing our cognition. World Psychiatry. (2019) 18:119–29. doi: 10.1002/wps.20617, 31059635 PMC6502424

[61] Zhang C Lin L Wang L Hu H Zhang H. The double-edged sword of digital engagement—how digital access and internet use reshape sleep schedules and underlying mechanisms in older adults: longitudinal observational study. JMIR Aging. (2025) 8:e79731. doi: 10.2196/79731, 41191871 PMC12588592

[62] Chen Y Zhang X Akaishi R. Exploring digital use, happiness, and loneliness in Japan with the experience sampling method. npj Mental Health Res. (2024) 3:63. doi: 10.1038/s44184-024-00108-4, 39643615 PMC11624198

[63] Risko EF Gilbert SJ. Cognitive offloading. Trends Cogn Sci. (2016) 20:676–88. doi: 10.1016/j.tics.2016.07.002, 27542527

[64] Vujic A Mowszowski L Meares S Batchelor J Naismith SL. Not all mentally stimulating activities are alike: insights from a 4-factor model and implications for late-life cognition. Aging Neuropsychol Cognit. (2023) 30:822–36. doi: 10.1080/13825585.2022.2094878, 35775824

[65] Shao X Zhu L Yang C Zhou Q Zhang F Wang Y . The effects of digital device use on psychological health and cognitive functions in Chinese older adults. Int J Geriatr Psychiatry. (2026) 41:e70222. doi: 10.1002/gps.70222, 42176345

[66] Hsu E-C Spaulding EM Jutkowitz E. Technology activities and cognitive trajectories among community-dwelling older adults: National Health and aging trends study. JMIR Aging. (2025) 8:e77227. doi: 10.2196/77227, 41289578 PMC12646554

[67] Du Y Niu Q Tan G Chao J Jin S Wang L. Digital engagement and cognitive function among older adults in China: cross-sectional questionnaire study and moderated mediation model analysis. J Med Internet Res. (2026) 28:e83955. doi: 10.2196/83955, 41556560 PMC12869153

[68] Kim YK Han SH. Internet use and cognitive functioning in later life: focus on asymmetric effects and contextual factors. Gerontologist. (2022) 62:425–35. doi: 10.1093/geront/gnab149, 34614179 PMC8963164

[69] Barrantes Cáceres R Cozzubo Chaparro A. Age for learning, age for teaching: the role of inter-generational, intra-household learning in internet use by older adults in Latin America. Inf Commun Soc. (2019) 22:250–66. doi: 10.1080/1369118X.2017.1371785

