# Supplementary material for: Beyond access: how heterogeneity of internet use differentially shapes cognitive function among Chinese older adults
Source: Front Public Health. 2026 Jul 20;14:1851159. doi: 10.3389/fpubh.2026.1851159 (PMC13429455; doi:10.3389/fpubh.2026.1851159)
Supplement: Supplementary file 1 [file Table_1.DOCX]

Supplementary Material

# Supplementary Tables

## Supplementary Table 1

Supplementary Table 1 Community development measurement indicators

| Variable name | Variable measurement |
| --- | --- |
| Local urbanization rate | The urbanization level of the city where the community is located |
| Local education level | The average years of schooling in the city where the community is located |
| Community healthcare accessibility | The average number of community medical services utilized, including home nursing, home medical visits, rehabilitation training, rental of rehabilitation aids, free health checkups, health record establishment, and health lectures |
| Community social capital | The average participation rate in community activities |

## Supplementary Table 2

## Supplementary Table 2 Covariates balance testing for propensity score matching

| Variables | Unmatched | Mean value | | Deviation % | Deviation  reduction ratio % | T-test | |
| --- | --- | --- | --- | --- | --- | --- | --- |
|  | Matched | Treated | Control |  |  | T-value | P > \|t\| |
| Age | U | 69.961 | 73.486 | -69.30 | 97.10 | -27.50 | 0.000 |
|  | M | 69.983 | 70.087 | -2.00 |  | -0.85 | 0.393 |
| Gender | U | 0.541 | 0.503 | 7.80 | 95.60 | 3.43 | 0.001 |
|  | M | 0.541 | 0.539 | 0.30 |  | 0.12 | 0.907 |
| Education | U | 2.696 | 1.978 | 83.40 | 97.70 | 37.32 | 0.000 |
|  | M | 2.689 | 2.706 | -1.90 |  | -0.63 | 0.529 |
| Marital status | U | 0.834 | 0.682 | 36.10 | 97.20 | 14.92 | 0.000 |
|  | M | 0.833 | 0.829 | 1.00 |  | 0.39 | 0.695 |
| Number of chronic diseases | U | 1.835 | 1.707 | 8.40 | 86.70 | 3.78 | 0.000 |
|  | M | 1.819 | 1.836 | -1.10 |  | -0.37 | 0.712 |
| IADL_S_ | U | 7.321 | 7.925 | -35.1 | 93.80 | -13.34 | 0.000 |
|  | M | 7.322 | 7.360 | -2.2 |  | -1.10 | 0.271 |
| Living arrangement | U | 0.300 | 0.397 | -20.50 | 99.70 | -8.87 | 0.000 |
|  | M | 0.301 | 0.302 | -0.10 |  | -0.02 | 0.981 |
| Pension insurance | U | 0.858 | 0.746 | 28.3 | 98.40 | 11.73 | 0.000 |
|  | M | 0.857 | 0.855 | 0.50 |  | 0.18 | 0.788 |
| Life satisfaction | U | 2.129 | 2.292 | -19.20 | 80.50 | -8.28 | 0.000 |
|  | M | 2.131 | 2.099 | 3.8 |  | 1.36 | 0.174 |
| Residence | U | 0.230 | 0.507 | -60.0 | 98.90 | -25.05 | 0.000 |
|  | M | 0.231 | 0.234 | -0.7 |  | -0.16 | 0.874 |

Note: This table presents the test results from radius matching (r=0.05)

## Supplementary Table 3

Supplementary Table 3 Propensity score matching regression results

| Variables | Cognitive function | |
| --- | --- | --- |
|  | B | SE |
| Internet adoption | 0.849^***^ | 0.128 |
| Control variables | Yes | |
| Individual fixed effects | Yes | |
| Year fixed effect | Yes | |
| R-squared | 0.835 | |
| N | 13390 | |

Note: *p < 0.05, **p < 0.01, ***p < 0.001.

## Supplementary Table 4

Supplementary Table 4 Attrition rates by baseline internet use status

| Internet use status | Baseline N | Completed follow-up N | Attrition N | Attrition rate (%) |
| --- | --- | --- | --- | --- |
| Users | 7,633 | 5,751 | 1,882 | 24.66 |
| Non-users | 1,268 | 1,049 | 219 | 17.27 |
| Total | 8,901 | 6,800 | 2,047 | 23.00 |

Note: χ² = 32.88, p < 0.001 for the comparison of attrition rates between users and non-users.

## Supplementary Table 5

Supplementary Table 5 Transition of internet use status between 2018 and 2020

| 2018 internet use status | 2020 non-uses | 2020 users | Total |
| --- | --- | --- | --- |
| Non-uses | 10498 | 1004 | 11,502 |
| Users | 464 | 1634 | 2,098 |
| Total | 10962 | 2638 | 13600 |
